# Comparison between intravenous lidocaine and dexamethasone in reducing postoperative sore throat after endotracheal extubation at Tikur Anbessa Specialized Hospital, Addis Ababa, Ethiopia; a prospective cohort study

**DOI:** 10.1186/s12871-024-02634-2

**Published:** 2024-07-29

**Authors:** Samuel Belay Ayalew, Tinbite Daniel, Hirbo Samuel, Amanuel Sisay Endeshaw, Habtu Tsehayu Bayu

**Affiliations:** 1 Department of Anesthesia, College of Medicine and Health Science, Bahir Dar University, Bahir Dar, Ethiopia; 2 Department of Anesthesia, College of Medicine and Health Science, Addis Ababa University, Addis Ababa, Ethiopia

**Keywords:** Postoperative sore throat, Endotracheal extubation, Dexamethasone, Lidocaine

## Abstract

**Background:**

Post-operative sore throat is the common complaint and uncomfortable side effect in patients receiving general anesthesia with endotracheal intubation. Drugs with analgesic and anti-inflammatory properties, like steroids and local anesthetics, are the best options for postoperative sore throat prophylaxis. Therefore, this study aimed to compare the effects of intravenous lidocaine and dexamethasone in reducing postoperative sore throat following endotracheal extubation at Tikur Anbessa Specialized Hospital, Addis Ababa, Ethiopia, from January 1 to March 30, 2023 G.C.

**Methods:**

A prospective cohort study was carried out at Tikur Anbessa Specialized Hospital. Data from 50 patients in the lidocaine (1.5 mg/kg), 50 in the dexamethasone (8 mg), and 49 in the control groups were analyzed. The data were collected using observation based on structured questionnaires. A systematic random sampling technique was applied to select respondents. The data were entered into EpiData version 4.6.0.6 and transferred to STATA version 17 statistical software for analysis. A comparison of continuous data among the groups were performed using a one-way ANOVA test for parametric data. The Kruskal–Wallis rank test was used for non-parametric data. Associations between variables were tested using chi-squared test, Fisher’s exact test, and binary logistic regression. Bivariable and multivariable logistic regression was used to determine degree of association.

**Results:**

The incidence of POST was 40%, 32%, and 57.1% in the lidocaine, dexamethasone, and control groups, respectively (*P* = .0356). Dexamethasone reduced the incidence of POST during the first 24 h (AOR: 0.374, 95% CI: 0.149–0.939). However, no difference was observed in the severity of POST at 3 h (*p* = 0.130), 6 h (*p* = 0.096), 12 h (*p* = 0.313), and 24 h (*p* = 0.525) of the post-extubation period among the three groups. IV lidocaine did not effectively reduce the incidence and severity of postoperative sore throat at different time intervals.

**Conclusion and recommendation:**

Intravenous dexamethasone is more effective than intravenous lidocaine in reducing the incidence of postoperative sore throat among the groups. Based on these findings, intravenous dexamethasone is recommended to decrease the incidence of postoperative sore throat.

**Supplementary Information:**

The online version contains supplementary material available at 10.1186/s12871-024-02634-2.

## Introduction

Postoperative sore throat (POST) is the nociceptive pain caused by an injury to the throat and tracheal mucosa after general anesthesia (GA) with endotracheal intubation (ETI) [1]. It results in discomfort and pain following extubation and surgery, hoarseness of voice, coughing, and sleep disturbances, makes them have more trouble swallowing and speaking as well as it extends hospital stay, which may cause patients to postpone returning to their regular routine activities [1–3]. It ranks as the sixth most unfavorable postoperative incident [4].

The incidence of POST and hoarseness is as high as 30% to 70% [5]. The high variability of POST incidence is caused by a vast variety of parameters involved in POST, including the kind of airway device, size of endotracheal tube (ETT), insertion technique and number of attempts, use or type of lubricant, ETT cuff pressure, duration of ETI, type of anesthetic drug provided, and patient factors [1, 6–13]. A recent systematic review and meta-analysis in Ethiopia showed the pooled incidence of POST was 40.48%, which is very high [14].

A POST following endotracheal extubation may develop for a variety of reasons, and its incidence varies depending on the technique used to control the airway [4]. The mechanistic basis for POST is believed to be mucosal dehydration, trauma during intubation, and airway irritation and inflammation brought on by pressure from the endotracheal tube cuff on the tracheal wall [4, 9, 15].

It has noticeable consequences for surgical and anesthesia outcomes; like debilitating throat pain following surgery, patient dissatisfaction and discomfort after surgery, inability to swallow for several days, bad memories of symptoms, delayed patients return to normal routine activities, delayed discharge from the hospital, insomnia, and memory impairment. All these results in poor postoperative outcomes and an increased economic burden. Furthermore, anesthesia care providers may not be aware of the incidence of sore throat in their practice area as many patients may not seek medical advice for POST [13, 16].

Numerous non-pharmacological or pharmaceutical techniques such as using the right size and type of ETT, tracheal tube lubrication, checking cuff pressure during surgery, aspirin, lignocaine spray or injection, dexamethasone, magnesium sulfate, ketamine gargling, and stellate ganglion blocking have been introduced to reduce the frequency and severity of POST [2, 17–20]. Most of these methods are limited in low-income countries like Ethiopia, and are not well practiced. However, administration of IV dexamethasone and Lidocaine is simple, effective, easily available, and practicable in the operating room [21, 22].

Awareness of the prophylactic effects of IV Lidocaine and dexamethasone to decrease the incidence and severity of POST following GA with ETI will allow health care providers to use these drugs to reduce postoperative morbidity and patient dissatisfaction, and improve a patient’s anesthesia experience. The incidence of POST has been found to vary with different patient and hospital characteristics, such as different pain threshold levels among different populations, different airway management techniques and interventions among the groups. Our patients’ pleasure and the delivery of high-quality care are of utmost significance. Therefore, this study aims to compare the prophylactic effects of IV lidocaine and dexamethasone in reducing POST following endotracheal extubation at TASH, Addis Ababa, Ethiopia.

## Methods and materials

### Study design and period

A hospital-based prospective cohort study was conducted from January 1 to March 30, 2023 at Tikur Anbessa Specialized Hospital, Addis Ababa, Ethiopia. This hospital is one of the largest tertiary referral hospital in the country and receives a diverse set of patients from all around Ethiopia, and the study drugs are practiced in day to day activity for an indicated surgical patients.

### Source population and study population

This study source population were all adult patients who underwent elective surgery under GA with ETI at TASH during the study period, and the study population were all eligible adult patients who met the inclusion criteria during the study period as exposed and non-exposed group.

### Eligibility criteria

During the study period, patients who undergoing elective surgery under GA with ETT without the study drugs as the non-exposed group, patients who received either IV lidocaine (1.5 mg/kg) or dexamethasone (8 mg) before 5–10 min of anesthesia induction as the exposed group, Patients age from 18–65 years, and ASA physical status I and II were included, and patients with recent or ongoing URTI, patients who had a preoperative sore throat, being smoker, patients who undergo surgeries on the oral cavity or oropharynx, obstetrics surgery, patients who had anticipated difficult intubation, patients who had taken combined lidocaine and dexamethasone or other steroid agents, intubation attempts > 2, unpredicted long duration of surgeries (> 4 h), and patients were transferred to the intensive care unit with ETT in situ were excluded.

### Variables of the study

The dependent variables of this study was incidence and severity of postoperative sore throat, and the independent variables were demographic characterstics (Age, Sex, BMI, ASA physical status), preemptive analgesia, types of surgery, surgical positioning, exposure status (non-exposed group, Dexamethasone group, and Lidocaine group), induction agents, Cormach-Lehane grading scheme for laryngoscopy, attempts of ETI, size of ETT, and duration of endotracheal intubation.

### Sample size determination and sampling techniques

The sample size was calculated using a priori power analysis (g*power version 3.1.9.7 statistical software) after obtaining the sample mean and standard deviation of POST among the groups from a study done in Nablus-Palestine [22]. Mean of group-1 (lidocaine group) = 1.85, mean of group-2 (dexamethasone group) = 1.71, mean of group-3 (control group) = 1.57 and Standard deviation with in each group = 0.43. An error probability of 5%, a power of 80%, the calculated effect size (0.266), and a 10% attrition rate were used to calculate the sample size. Therefore, the sample size was 141 and adding a 10% attrition rate = 156. Subjects were selected for the study in an equal ratio to the groups (1:1:1 ratio), therefore, 52 patients were observed in each group.

The study subjects were chosen through a systematic random sampling method. As a sampling frame, the daily operation schedule list was employed. According to the situational analysis at TASH, 260 patients who meet the inclusion criteria had surgery from September 3- to- November 2, as determined by surgical log book registrations. Therefore, the sample was collected using a sample interval (k) = 156/260. K value was determined to be 3/5 from this sampling fraction, and data collection was conducted on three patients for every five patients who undergo surgery under GA with ETT. The study’s first patient was randomly selected, and the subsequent patients were arranged in the order in which they were admitted to the PACU.

### Data collection techniques and quality control

After training the data collectors (two BSc and four MSc anesthesia students), the data was collected and properly filled out in the pre-designed format. Based on data from a chart review and patient interview, all patients who were scheduled for elective surgery under GA with ETT and met the inclusion criteria were selected at the preoperative period. All patients were managed with standard perioperative monitoring’s by MSc anesthetists and anesthesiologists. Data about the demographic, surgical, and perioperative anesthesia profiles; including age, gender, BMI, ASA physical status, types of surgery, preemptive analgesia, surgical positioning, induction agents, Laryngoscopic grades, number of attempts of ETI, size of ETT, and duration of ETI, were observed and filled out by anesthetist data collectors. Surgical patients who received 8 mg dexamethasone or 1.5 mg/kg lidocaine intravenously before 5-to-10 min of anesthesia induction as the exposed group and those who did not receive both IV lidocaine and dexamethasone as the non-exposed group (but taken other anesthesia and surgical treatments as an exposed groups) were recorded by the responsible anesthetist data collectors.

All the study subjects were induced with IV induction agents and relaxed with 2 mg/kg of succinylcholine during ETI. In the intraoperative period, all patients were ventilated with oxygen and maintained anesthesia with inhalational anesthetic drugs and 0.1 mg/kg of Vecronium for relaxants. At the end of surgery, muscle relaxation was reversed by 0.05 mg/kg neostigmine and 0.02 mg/kg atropine. The patients were extubated after fulfilling the extubation criteria and transferred to the PACU and then to the ward. The accountable data collector, who was not informed of the group allocation by using codes to represent the groups, gathered the postoperative data. Postoperative follow-up and data collection on the incidence and severity of POST and postoperative analgesia consumption were collected at the surgical ward for 24 h. Data collection was started at 3 h after being transferred to PACU, then continued at 6 h, 12 h, and 24 h.

### Data analysis

The data were manually reviewed for completeness and then coded and entered into EpiData version 4.6.0.6 and transferred to STATA version 17 statistical software for analysis. The Shapiro–Wilk test and histogram were used to determine the normality of the data distribution for continuous data. Multicollinearity was checked using variance inflation factors (VIF) with a tolerance of 10% to describe categorical variables. Goodness of fit test was also checked using Hosmer and Lemeshow test. A comparison of parametric data among the groups such as age and duration of ETI was performed using a one-way ANOVA test. The Kruskal–Wallis rank test was applied for non-parametric data such as BMI. Associations between independent and dependent variables were tested by using chi-squared test, Fisher’s exact test, and binary logistic regression. Bivariable and multivariable logistic regression was used to determine degree of association. The findings of continuous variables were expressed as mean ± SD, and categorical variables has been presented as frequencies and percentages. Finally, the variables that have a significant association with the outcome variable was expressed using odds ratio, 95% CI, and *p*-value of less than 0.05 was considered as statistically significant.

### Operational definition

*Postoperative sore throat*: considered present if the patient complains of pain, scratchiness, or irritation of the throat at the postoperative period. Therefore, the data was recorded as yes if POST occurred within 24 h of post-extubation period and no if it was not occurred within the mentioned time range. The patients graded the severity of a sore throat using the scoring system as described previously [23, 24] which can be stated verbally.

The degree of sore throat is represented by the following pain assessment tool.

- 0 = No sore throat.
- 1 = Minimal sore throat (complaints of sore throat only on asking).
- 2 = Moderate sore throat (complaints of sore throat on his/her own).
- 3 = Severe sore throat (change in voice or hoarseness, associated with throat pain) over 24 h.

**Fig. 1:**
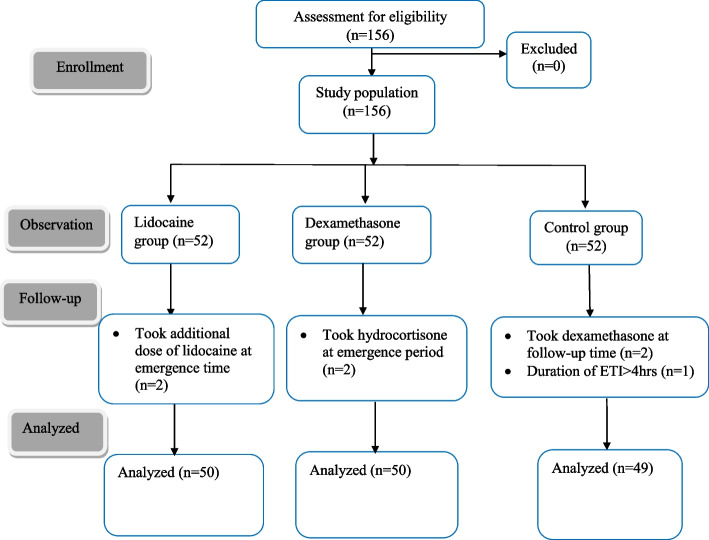
Flow diagram of sampling of study participants

The required number of samples for each group was identified as follows (Fig. 1).

## Results

### Demographic and clinical parameters related to the study groups

Among a sample of 156 patients enrolled in this study, 149 were included in the final analysis. Seven patients were excluded from the analysis: two had received an additional dose of lidocaine at the time of emergence, two had received hydrocortisone at the time of emergence, two had received dexamethasone at the time of follow-up, and one patient had a prolonged duration of surgery (> 4 h). The results indicate that there was no significant difference among the three groups in both demographic data, surgical characteristics, and perioperative profiles of the patient (Table 1).

**Table 1:** Demographic and clinical profiles of the study subject among lidocaine, dexamethasone and control groups

Characterstics	Lidocaine group (*n* = 50)	Dexamethasone group (*n* = 50)	Control group (*n* = 49)	*p*-value
Age (yrs.)^a^	40.78 ± 8.087	40.48 ± 10.124	40.59 ± 9.246	.986
Gender^c^				.589
Male	25(50%)	20(40%)	23(47%)	
Female	25(50%)	30(60%)	26(53%)	
BMI (kg/m^2^)^b^	20.6(4.1)	19.85(4.5)	20.9(4.1)	.1287
ASA physical status^c^				.683
ASA I	20 (40%)	24(48%)	23(47%)	
ASA II	30(60%)	26(52%)	26(53%)	
Types of surgery^c^				.284
Abdominal	9(18%)	7(14%)	4(8.2%)	
Urology	11(22%)	12(24%)	8(16.3%)	
Endocrine	10(20%)	12(24%)	12(24.5%)	
Vascular	8(16%)	3(6%)	4(8.2%)	
Orthopedics	7(14%)	7(14%)	16(32.6%)	
Gynecology	5(10%)	9(18%)	5(10.2%)	
Surgical positioning^c^				.407
Supine	30(60%)	35(70%)	33(67.3%)	
“Supine with head tilt”	11(22%)	5(10%)	5(10.2%)	
Lateral	9(18%)	10(20%)	11(22.5%)	
Preemptive analgesia^c^				.486
Fentanyl	37(74%)	43(86%)	35(71.4%)	
Morphine	9(18%)	6(12%)	8(16.3%)	
Tramadol	2(2%)	1(2%)	4(8.2%)	
Diclofenac	2(4%)	0(0%)	2(4.1%)	
Induction agents^c^				.347
Ketamine	4(8%)	3(6%)	1(2%)	
Propofol	32(64%)	31(62%)	34(69.4%)	
Ketofole	8(16%)	12(24%)	5(10.2%)	
Thiopentone	6(12%)	4(8%)	9(18.4%)	
Laryngoscope grade^c^				.355
Grade-1	36(72%)	40(80%)	33(67.3%)	
Grade-2	14(28%)	10(20%)	16(32.7%)	
Attempts of ETI^c^				.285
One-attempt	49(98%)	49(98%)	45(91.8%)	
Two-attempts	1(2%)	1(2%)	4(8.2%)	
Size of ETT^c^				.376
6.0	10(20%)	15(30%)	8(16.3%)	
6.5	14(28%)	16(32%)	20(40.8%)	
7.0	20(40%)	15(30%)	13(26.6%)	
7.5	6(12%)	4(8%)	8(16.3%)	
Duration of ETI(min)^a^	161 ± 33	154 ± 35	151 ± 28	.328
“Post-op analgesia consumption”^c^				.166
Diclofenac	4(8%)	3(6%)	11(22.5%)	
Tramadol	6(12%)	2(4%)	3(6.1%)	
“Diclofenac + tramadol”	36(72%)	42(84%)	31(63.3%)	
Morphine	2(4%)	1(2%)	3(6.1%)	
Paracitamol	2(45)	2(4%)	1(2%)	

^a^mean ± SD, tested by one-way ANOVA

^b^median (IQR), tested by the Kruskal–Wallis rank test

^c^number (%), tested by the chi-square test and Fisher’s exact test

### Incidence and severity of postoperative sore throat among lidocaine, dexamethasone and control groups

**Fig. 2:**
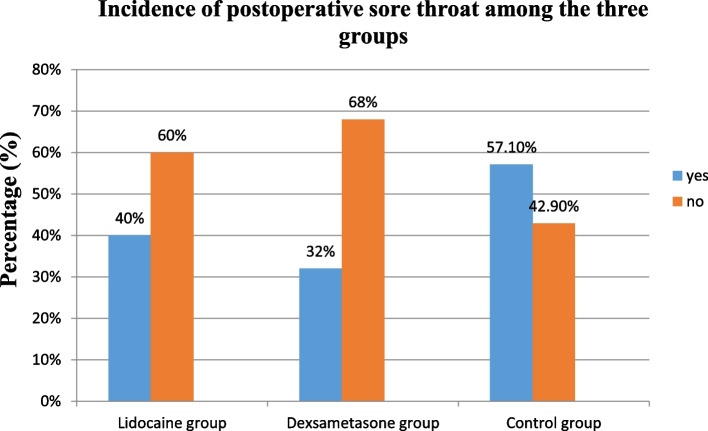
Incidence of POST during 24 h of post-extubation period among the three groups

The incidence of POST within 24 h of the post-extubation period in the Lidocaine, Dexamethasone, and Control groups was 40%, 32%, and 57.1%, respectively. It shows an association between lidocaine, dexamethasone, and control groups relating to the incidence of POST following tracheal extubation (*p* = 0.036) (Fig. 2).

Based on chi square and Fisher’s exact test analysis, there was no association between lidocaine, dexamethasone, and control groups relating to the severity of POST at different time intervals during 24 h of post extubation periods (*p* = 0.130, 0.096, 0.313, and 0.525 at 3 h, 6 h, 12 h, and 24 h respectively) (Table 2).

**Table 2:** Severity of postoperative sore throat among the three groups

Severity of POST at different time intervals	Scales of POST	Lidocaine group (*n* = 50)	Dexamethasone group (*n* = 50)	Control group (*n* = 50)	*p*-value
Severity of POST at 3 h	No POST	30(60%)	34(68%)	21(42.9%)	.130
	Minimal POST	15(30%)	12(24%)	17(34.7%)	
	Moderate POST	5(10%)	3(6%)	8(16.3%)	
	Sever POST	0(0%)	1(2%)	3(6.1%)	
Severity of POST at 6 h	No POST	30(60%)	35(70%)	21(42.9%)	.096
	Minimal POST	16(32%)	11(22%)	18(36.7%)	
	Moderate POST	4(8%)	3(6%)	7(14.3%)	
	Sever POST	0(0%)	1(2%)	3(6.1%)	
Severity of POST at 12 h	No POST	37(74%)	38(76%)	28(57.2%)	.313
	Minimal POST	11(22%)	9(18%)	15(30.6%)	
	Moderate POST	2(4%)	3(6%)	5(10.2%)	
	Sever POST	0(0%)	0(0%)	1(2%)	
Severity of POST at 24 h	No POST	41(82%)	42(84%)	34(69.4%)	.525
	Minimal POST	8(16%)	7(14%)	11(22.5%)	
	Moderate POST	1(2%)	1(2%)	3(6.1%)	
	Sever POST	0(0%)	0(0%)	1(2%)	

^*^number (%), tested by the chi-square test and Fisher’s exact test

### Interpretation of multivariable logistic regression in terms of adjusted odds ratio

In a bivariable study, gender, preemptive analgesia, surgical positioning, induction agents, attempts at ETI, lidocaine, and dexamethasone groups were found to be positively associated with the incidence of POST with a *p*-value of less than 0.25 and were candidates for multivariable logistic regression.

In multivariate logistic regression, the incidence of POST in elective adult surgical patients following endotracheal extubation was significantly associated with gender, surgical positioning, and dexamethasone group.

Patients of female gender were 2.7 times (AOR: 2.69, 95% CI: 1.26, 5.73) more likely to develop POST than males. The odds of developing POST were 5 times (AOR: 5.03, 95% CI: 1.66–15.26) more in patients who underwent surgery using supine with head tilt positioning than those patients who underwent surgery with supine and lateral positions. Dexamethasone group had a marked reduction in the incidence of POST by 63% (AOR: 0.37, 95% CI: 0.15–0.94) (Table 3).

**Table 3:** Bivariable and Multivariable binary logistic regression-shows factors associated with the incidence of POST among elective surgical patients at TASH

Variables	Categories	Incidence of POST		COR(95% CI)	AOR(95% CI)	*P*-value
		Yes (*n* = 64)	No (*n* = 85)			
Gender	Male	22	46	1	1	
	Female	42	39	2.25(1.15–4.34)	2.69(1.26–5.73)	0.010*
Preemptive analgesia	Fentanyl	47	68	1	1	
	Morphine	9	14	0.93(0.37–2.33)	0.86(0.30–2.50)	0.787
	Tramadol	5	2	3.62(0.67–19.44)	3.72(0.59–23.71)	0.164
	Diclofenac	3	1	4.34(0.44–43.01)	4.80(0.39–5.96)	0.223
Surgical positioning	Supine	34	64	1	1	
	Supine with head tilt	15	6	4.71(1.67–13.24)	5.03(1.66–15.26)	0.004*
	Lateral	15	15	1.88(0.82–4.31)	2.13(0.84–5.41)	0.111
Induction agents	Ketamine	2	6	1	1	
	Propofol	42	55	2.29(0.44–11.93)	1.01(0.18–5.80)	0.987
	Ketofole	9	16	1.69(0.28–10.17)	0.92(0.14–6.15)	0.931
	Thiopentone	11	8	4.13(0.65–26.01)	1.66(0.24–11.55)	0.611
Attempts of ETT	One attempt	60	83	1	1	
	Two attempt	4	2	2.77(0.49–15.60)	3.09(0.43–22.21)	0.262
Groups of drugs	Lidocaine	20	30	0.50(0.23–1.11)	0.45(0.18–1.11)	0.084
	Dexamethasone	16	34	0.035(0.16–0.80)	0.37(0.15–0.94)	0.036*
	Control	28	21	1	1	

*COR* Crude odds ratio, *AOR* Adjusted odds ratio, *CI* Confidence interval, *1* Reference

^*^Significant in the Multivariable binary logistic regression (*p*-value < 0.05)

## Discussion

This study compared the effects of IV lidocaine and dexamethasone in reducing POST in patients undergoing elective surgery under GA with ETT. The incidence of POST during the first 24 h was 32%, 40%, and 57.1% in the dexamethasone, lidocaine, and control groups, respectively. This finding has been consistent with a study done by Subedi et al. that showed the incidence of POST in patients requiring GA with ETT was 36%, 43%, and 56% in the dexamethasone, lidocaine, and normal saline groups, respectively [4].

This present study demonstrates that the incidence of POST within the first 24 h of post-extubation period was lower in the dexamethasone group than the control group by 63% (AOR: 0.37, 95% CI: 0.15–0.94). This result is consistent with studies carried out in Iran, Palestine, and India that found IV dexamethasone was effective in reducing the incidence of POST in patients requiring ETI [4, 20, 22]. The possible reasons are due to its anti-inflammatory and immunosuppressive properties, which are explained by central inhibition of prostaglandin synthesis, decreased central nervous system serotonin turnover, and modulation of the systemic inflammatory response in favor of its anti-inflammatory mediators [25, 26].

Similar to this present study, a double-blind randomized trial conducted in Korea and Tanzania found that patients who received IV dexamethasone prior to induction of anesthesia experienced significantly lower rates of POST than those who received placebo at 1, 6, and 24 h after tracheal extubation [25, 27]. This similar finding is probably due to the use of a similar dose of dexamethasone and an approximate sample size. This study was also comparable to a meta-analysis of randomized controlled trials conducted in Ireland by L. Sun. et al., which showed that a single dose of intravenous dexamethasone reduced the incidence of POST within 24 h of surgery [28].

However, research conducted by Ruangsin et al. revealed that there was no significant difference in the effectiveness of two different doses of prophylactic IV dexamethasone (4 mg and 8 mg) against POST following ETI [29]. This contradictory finding is probably due to the relatively small sample size (35 in each group) and different analytic models; they used the unpaired Student’s t-test and Mann–Whitney U test for the analysis of continuous data among the three groups (4 mg dexamethasone group, 8 mg dexamethasone group, and 2 ml normal saline group).

In this present study, prophylactic IV lidocaine (1.5 mg/kg) did not significantly decrease the incidence of POST after endotracheal extubation in patients who underwent elective surgery under GA with ETT. This is comparable to a randomized controlled trial conducted in India and Palestine that revealed that IV lidocaine alone was ineffective in reducing POST following surgery [4, 29].

On contrary to this study, a meta-analysis of systematic reviews conducted by Tanaka et al. in 2015 revealed that lidocaine administered topically and intravenously significantly decreased the risk of sore throat after surgery. However, a subgroup analysis of high-quality studies shows that there are no statistically significant differences in preventing POST related to IV lidocaine [5]. This contradictory finding is probably due to a different study design; they were doing a scientific review study.

According to the results of the current study, we found that giving prophylactic IV dexamethasone or lidocaine to patients undergoing elective surgery under GA with ETT can’t have an association with the severity of POST at different time intervals (3 h, 6 h, 12 h and 24 h) after endotracheal tube extubation. This result is supported by a study conducted in India, which suggested that there were no significant differences in the severity of POST among the groups at 12 and 24 h following surgery [4]. Similar findings have been observed in studies conducted by Yang C. et al. showed that IV injection of prophylactic 10 mg dexamethasone did not have a significant effect on the severity of POST at rest or while swallowing in the first 6 h after endotracheal extubation [30].

Contrary to this finding, a study conducted by Mohan et al. and Thomas et al. that show IV dexamethasone before surgery decreases the severity of POST after endotracheal tube extubation at 1 h, 3 h, and 6 h following surgery [26, 31]. This finding is also contrary to a study done by Nandi et al., which found that prophylactic IV dexamethasone reduces the severity of sore throat after ETI at 1 h and 6 h after surgery [25]. This difference is probably due to using of different methodology and different target populations.

According to the findings of this study, the variables significantly associated with the incidence of POST were female gender and supine with head tilt surgical positioning. Female patients were 2.7 times more likely to develop POST than males. This finding is in line with studies conducted in Debre Tabor General Hospital, north-central Ethiopia showed that female patients were significantly associated to POST with an odds of 2.58 times more risky than males [6]. Another similar studies conducted in Gondar, Ethiopia and south Ethiopia also supports the findings of this research [7, 14]. One of the causes of this difference is that due to reporting bias because women’s are more likely to report such postoperative side effects [32]. In contrary to this study, a research conducted at Nepal found that there were no significant association between sex and postoperative sore throat following tracheal extubation [4].

In this current study, the odds of developing a postoperative sore throat after surgery in supine with head tilt surgical positioning were 5 times greater than those of patients who underwent surgery in supine and lateral positions. The probable mechanism could be that the endotracheal tube may move according to the patient’s position, which may have an impact on the frequency of POST [27].

### Strengths of the study

Baseline variables, including demographic, surgical, and perioperative characteristics, were homogeneous. The sample size is adequate, and the response rate is high. Experienced anesthesia staff were involved in data collection, which adds to the reliability of the data. This study addresses an important clinical issue and provides valuable insights for clinical practice and future research on the prevention of POST; since published research could not be found in our country, Ethiopia.

### Limitations of the study

Tracheal cuff pressures were not monitored in this present study due to lack of equipment; hence, sore throat may develop as a result of high cuff pressures during anesthesia by causing mucosal ischemia [8]. As it is an observational study, certain confounding factors could not be controlled. Another limitation is that the study was conducted at a single center, which may limit the generalizability of the findings to the regions.

## Conclusion

Intravenous dexamethasone (8 mg) decreased the incidence of POST during the first 24 h of the post-extubation period in patients who underwent elective surgery under GA with ETI. However, no difference was observed in the severity of POST at different time intervals (3 h, 6 h, 12 h and 24 h of post-extubation period) among lidocaine, dexamethasone and control groups. Intravenous lidocaine (1.5 mg/kg) could not have significantly reduced the incidence and severity of POST at different time intervals. Female gender and supine with head tilt surgical positioning were the variables significantly associated with the incidence of POST.

### Recommendations

The incidence and severity of postoperative sore throat is high, so we suggest prophylactic intravenous dexamethasone be given to patients who underwent elective surgery under GA with ETT to decrease the incidence of POST.

### Supplementary Information

Supplementary Material 1.

## Data Availability

No datasets were generated or analysed during the current study.

## Abbreviations

AAU: Addis Ababa University
ANOVA: Analysis of Variance
AOR: Adjusted Odds Ratio
ASA: American Society of Anesthesiologists
COR: Crude Odds Ratio
ETI: Endotracheal Intubation
ETT: Endotracheal Tube
GA: General Anesthesia
IV: Intravenous
POST: Postoperative Sore Throat
STATA: Statistics and Data
TASH: Tikur Anbessa Specialized Hospital
