# Supplementary material for: Comparison between intravenous lidocaine and dexamethasone in reducing postoperative sore throat after endotracheal extubation at Tikur Anbessa Specialized Hospital, Addis Ababa, Ethiopia; a prospective cohort study
Source: BMC Anesthesiol. 2024 Jul 29;24:259. doi: 10.1186/s12871-024-02634-2 (PMC11285459; doi:10.1186/s12871-024-02634-2)
Supplement: Supplementary file 1 — Supplementary Material 1. [file 12871_2024_2634_MOESM1_ESM.pdf]

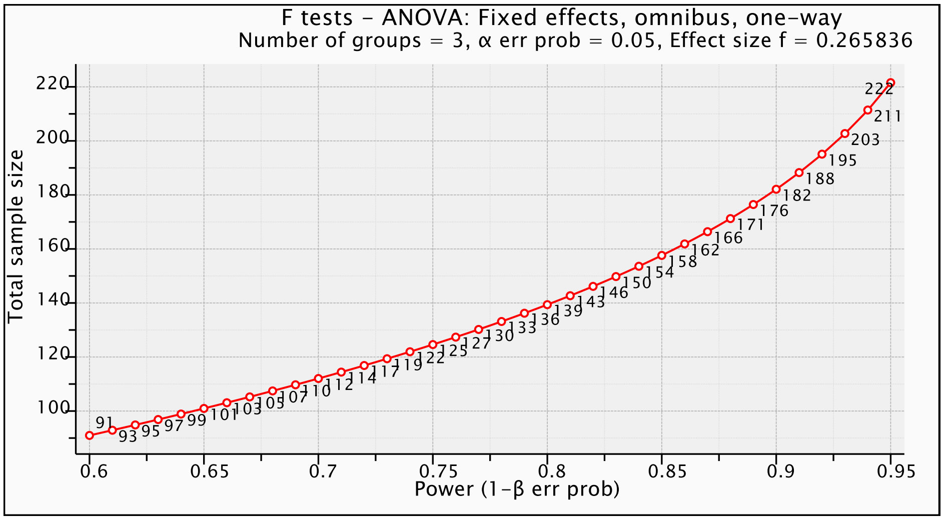

Supplementary Figure Sample size determination using g*power software

Questionnaire

A data collection format for adult patients who underwent elective surgery at TASH, Addis Ababa, Ethiopia. Fill in the blank space provided, encircle the alternatives when necessary, and finally check the questions for completeness.

1. Demographic data

101	Patient code number	……….(please write this code number on the patient's card)
102	Age	..……..…years
103	Gender	1. Male<br>2. Female
104	Weight	………kg
105	BMI	………kg/m^2
106	ASA physical status	1. I<br>2. II<br>3. ≥ III

2. Preoperative and intraoperative anesthesia and surgical interventions

201	Types of surgery	1. Abdominal<br>2. Urology<br>3. Endocrine	4. Vascular<br>5. Orthopedic<br>6. Gynecology
202	Preemptive Analgesia given (You can answer more than one alternative)	1. Fentanyl………..mcg<br>2. Pethidine……….mg<br>3. Morphine……….mg<br>4. Tramadol……….mg	5. Non-opioid analgesia (specify with dose)……….<br>6. Not premedicated
203	Surgical positioning of the patient	1. Supine<br>2. Trendelenburg<br>3. Reverse trendelenburg	4. Lateral<br>5. Prone<br>6. Lithotomy
204	Groups (which pre-medication agent is taken	1. Lidocaine……………..……mg IV<br>2. Dexamethasone…………….mg IV	

	to decrease postoperative sore throat?)	3. Neither of the two (control group)<br>4. Combined Lidocaine & dexamethasone	
205	Induction agents	1. Ketamine<br>2. Propofol	3. Ketofole<br>4. Thiopentone
206	Cormack-Lehane grading scheme for laryngoscopy	1. I<br>2. II	3. III<br>4. IV
207	Attempts of Endotracheal Tube Intubation	1. 1<br>2. 2<br>3. >2	
208	Size of ETT	…………….mmID	
209	Types of ETT cuffs	1. High-volume, low-pressure<br>2. High-pressure, low-volume	
210	Does the patient take additional lidocaine before extubation?	1. Yes (specify the dose)………..mg<br>2. No	
211	Does the patient take additional dexamethasone before extubation?	1. Yes (specify the dose)………..mg<br>2. No	
212	Duration of intubation	………………in minute	
213	Is the patient extubated in the operation theater?	1. Yes<br>2. No	

3. Postoperative sore throat characterstics

301	Do you have sore throat at 3-hours of the postoperative period?	1. No sore throat (0)<br>2. Minimal sore throat (1)<br>3. Moderate sore throat (2)<br>4. Sever sore throat (3)

302	Do you have sore throat at 6-hours of the postoperative period?	1. No sore throat (0)<br>2. Minimal sore throat (1)<br>3. Moderate sore throat (2)<br>4. Sever sore throat (3)
303	Do you have sore throat at 12-hours of the postoperative period?	1. No sore throat (0)<br>2. Minimal sore throat (1)<br>3. Moderate sore throat (2)<br>4. Sever sore throat (3)
304	Do you have sore throat at 24-hours of the postoperative period?	1. No sore throat (0)<br>2. Minimal sore throat (1)<br>3. Moderate sore throat (2)<br>4. Sever sore throat (3)
305	Postoperative total analgesia consumption	Specify……………………..

Postoperative sore throat assessment tools

A. The scale was taken four times during 24 hours of the post-extubation period following surgery.

B. Patients were asked the following questions to rate the severity of their sore throat:

1. What number on a 0 to 3 scale would you give your sore throat right now?
2. When the explanation suggested above is not sufficient for the patient, further explanation of the scale is done:

    0 = No sore throat

    1 = Minimal sore throat (complaints of sore throat only on asking)

    2 = Moderate sore throat (complaints of sore throat on his/her own)

    3 = Severe sore throat (change in voice or hoarseness, associated with throat pain)

Amharic version of postoperative sore throat assessment tools

ከቀዶ ህክምና በኋላ የጉሮሮ ህመም መለኪያ

1. ይህ መለኪያ ከቀዶ ህክምና በኋላ የመጀመሪያው 24 ሰዐት 4 ጊዜ የሚወሰድ ይሆናል።
2. ስለ ታካሚዉ ጉሮሮ ህመም መጠን ለማወቅ የሚከተሉትን መጠይቆች ይጠየቃል።

ሀ. ታካሚው የሚጠየቃቸው ጥያቄዎች

i. አሁን የሚሰማዎትን ህመም በየትኛው ቁጥር ይወክላሉ?

ii. ከ 0 እስከ 3 ካሉት ቁጥሮች አሁን የሚሰማዎትን ህመም የትኛው ቁጥር ይገልጸዋል?

ለ. ከላይ የተስጠው ማብራሪያ በቂ ሳይሆን ሲቀር፡ ለታካሚው የበለጠ መረጃ መስጠት አስፈላጊ ሆኖ ይገኛል።

i. 0 → ምንም ህመም የለም

ii. 1 → ትንሽ ህመም አለ

iii. 2 → መካከለኛ ህመም አለ

iv. 3 → ከባድ ህመም አለ
